# Early DNase-I therapy delays secondary brain damage after traumatic brain injury in adult mice

**DOI:** 10.1038/s41598-023-30421-5

**Published:** 2023-03-16

**Authors:** Tobias J. Krämer, Florian Pickart, Bruno Pöttker, Christina Gölz, Axel Neulen, Tobias Pantel, Hermann Goetz, Katharina Ritter, Michael K. E. Schäfer, Serge C. Thal

**Affiliations:** 1 Department of Anesthesiology, University Medical Center of Johannes Gutenberg University, Langenbeckstrasse 1, 55131 Mainz, Germany; 2 Faculty of Health, University Witten/Herdecke, Witten, Germany; 3 Department of Neurosurgery, University Medical Center of Johannes Gutenberg University, Langenbeckstrasse 1, 55131 Mainz, Germany; 4 Cell Biology Unit, University Medical Center of Johannes Gutenberg University, Langenbeckstrasse 1, 55131 Mainz, Germany; 5 Focus Program Translational Neurosciences, University Medical Center of Johannes Gutenberg University, Langenbeckstrasse 1, 55131 Mainz, Germany; 6 Research Center for Immunotherapy, University Medical Center of Johannes Gutenberg University, Langenbeckstrasse 1, 55131 Mainz, Germany; 7 Center for Molecular Surgical Research, University Medical Center of Johannes Gutenberg University, Langenbeckstrasse 1, 55131 Mainz, Germany; 8 Department of Anesthesiology, Helios University Hospital Wuppertal, University Witten/Herdecke, Heusnerstrasse 40, 42283 Wuppertal, Germany

**Keywords:** Neuroscience, Medical research, Neurology

## Abstract

Traumatic brain injury (TBI) causes the release of danger-associated molecular patterns (DAMP) from damaged or dead cells, which contribute to secondary brain damage after TBI. Cell-free DNA (cfDNA) is a DAMP known to cause disruption of the blood–brain barrier (BBB), promote procoagulant processes, brain edema, and neuroinflammation. This study tested the hypothesis that administration of deoxyribonuclease-I (DNase-I) has a beneficial effect after TBI. Mice (n = 84) were subjected to controlled cortical impact (CCI) and posttraumatic intraperitoneal injections of low dose (LD) or high dose (HD) of DNase-I or vehicle solution at 30 min and 12 h after CCI. LD was most effective to reduce lesion volume (*p* = 0.003), brain water content (*p* < 0.0001) and to stabilize BBB integrity (*p* = 0.019) 1 day post-injury (dpi). At 6 h post injury LD-treated animals showed less cleavage of fibrin (*p* = 0.0014), and enhanced perfusion as assessed by micro-computer-tomography (*p* = 0.027). At 5 dpi the number of Iba1-positive cells (*p* = 0.037) were reduced, but the number of CD45-positive cells, motoric function and brain lesion volume was not different. Posttraumatic-treatment with DNase-I therefore stabilizes the BBB, reduces the formation of brain edema, immune response, and delays secondary brain damage. DNase-I might be a new approach to extend the treatment window after TBI.

## Introduction

Traumatic brain injury (TBI) is the most frequent cause of death and disability among all trauma-related injuries worldwide. An estimated 5.5 million people suffer each year a severe traumatic brain injury (73 cases per 100,000 people). WHO estimates that nearly 90% of injury related deaths occur in developing countries^1,2^.

Damage-associated molecular patterns (DAMPs) are released from injured cells into surrounding tissue and systemic circulation in the early phase after TBI. DAMPs give rise to sterile systemic inflammatory response^3^, blood–brain-barrier (BBB) disruption^4^ and are associated with increased coagulopathy and mortality in TBI^5^, endotheliopathy after major trauma^6^ and inflammation during ischemic stroke^7^. One of the central components of DAMPs are cell-free deoxyribonucleic acids (cfDNA). cfDNA plasma levels are increased after TBI, which induces deoxyribonuclease-1 (DNase-I) activity, both effects being the focus of research as predictive markers for a severe course after TBI^5,8–11^. Thus, cfDNA has the potential to be more than a diagnostic tool, it is actively involved in increasing lesion volume after TBI, and more detailed investigations could lead to a new treatment approach. Moreover, activated neutrophils result in the formation of neutrophil extracellular traps (NETs) after TBI^12^, cerebral ischemia^13^, and also in COVID-19 acute respiratory distress syndrome^14^. NET formation co-occurred with cerebral hypoperfusion and tissue hypoxia after experimental TBI, and elevated circulating NETs correlated with reduced serum DNase-I activity in patients with TBI^12^. NETs are also released by microglial cells^15^ and physiologically degraded by endogenous nucleases such as DNase-I. Therefore, application of DNase-I might be a therapeutic approach to reduce inflammation and improve perfusion following TBI. Pulmozyme^®^, also known as dornase alfa, is a commercially available recombinant human DNase-I that is approved for the treatment of cystic fibrosis^16^ and currently used in a prospective, randomized, multicenter, double-blinded, placebo-controlled clinical trial to reduce the incidence of moderate-to-severe hypoxemia in ventilated trauma patients^17^ and also diminished NETs formation in COVID-19 patients^18^. Therapeutic treatment with DNase-I has also been examined in multiple animals studies and improved motor and psychiatric function at 2 months after controlled cortical-impact (CCI)^12^, and improved pericontusional perfusion after stroke in mice^19^.

This study was designed to investigate the effect of DNase-I treatment on inflammation, blood coagulation and BBB leakage after TBI. All three pathological events play a major role in the pathophysiology of TBI. Neuroprotective drugs should prevent the expansion of the primary injury into the healthy surrounding tissue. DNase-I could be a promising tool to suppress these crucial mechanisms. To this end, the influence of DNase-I treatment on functional and histological outcome as well as on the BBB, edema formation, influence on the immune system and blood coagulation at an early stage 1 day after lesion (1 dpi) and an intermediate stage 5 days after lesion (5 dpi) was investigated in the CCI model of TBI in mice.

## Results

### Influence of DNase-I treatment on brain damage after controlled cortical impact (CCI)

**Figure 1:**
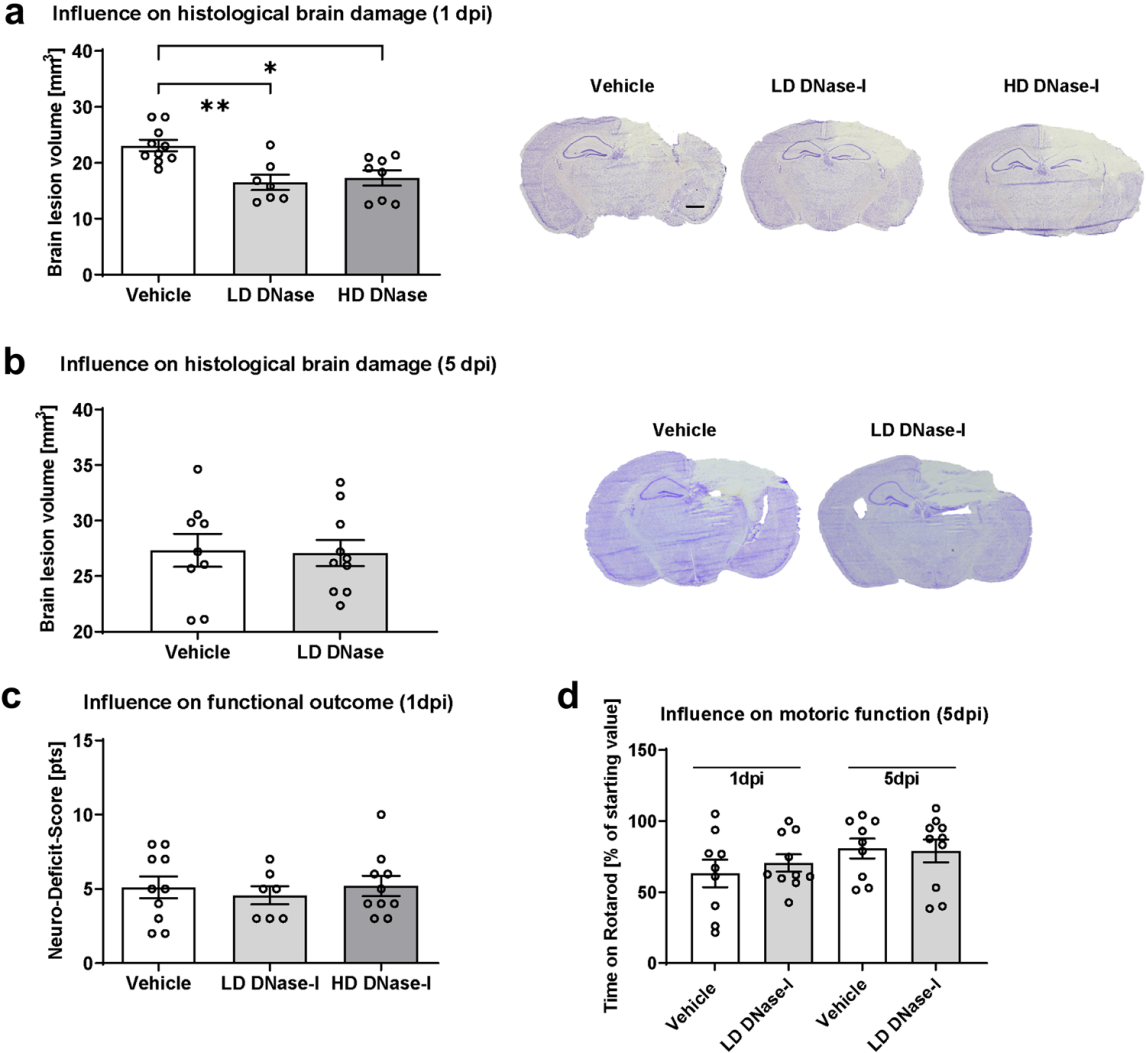
Effects of DNase-I injection on lesion size, neurological and motoric deficit after traumatic brain injury (TBI). (**a**) Brain lesion volume was determined 24 h (1 dpi) after controlled cortical impact (CCI) in mice treated with DNase-I low-dose or high dose (LD: 2 or HD: 18 m/kg) or vehicle (NaCl 0.9%) (n = 10 mice/group) by intraperitoneal injection 30 min and 12 h post-insult. Both DNase-I doses significantly reduced brain lesion volume compared to vehicle at 1 dpi. (**b**) Lesion volume did not differ between groups at 120 h post-CCI (5 dpi). (**c**) Neurofunctional deficit score at 1 dpi did not differ among groups. (**d**) Motor function as assessed by rotarod performance was severely impaired 1 dpi but recovered markedly by 5 dpi without significant difference between treatment groups (n = 12 mice/group). A significance level p < 0.05 is marked with *, p < 0.01 with **, and p < 0.001 with ***. All data are presented as mean ± SEM; *p* values are adjusted for multiple comparisons by Sidak correction.

At 1 dpi, brain lesion volume was quantified in cresyl violet-stained sections. The DNase-I treated animals showed a significant reduction of lesion size compared to Vehicle at 1 dpi (Fig. 1a, results as mean ± SEM: Vehicle 23.10 ± 1.02 mm^3^, LD-DNase-I 16.50 ± 1.39 mm^3^, HD-DNase-I 17.28 ± 1.37 mm^3^; Vehicle vs. LD-DNase-I *p* = 0.003; Vehicle vs. HD-DNase-I *p* = 0.012; n = 10/group). To test the consequences of the more effective LD-DNase-I on brain damage progression, brain lesion volume was also examined at 5 dpi. However, LD DNase-I treatment delayed, but did not prevent the secondary lesion growth as no difference was observed between the treatment groups regarding lesion volume at 5 dpi (Fig. 1b).

### Influence of DNase-I treatment on functional outcome

In addition to assessing lesion volume, we quantified the impact on functional outcome. For this purpose, functional impairment was tested with a neuro-deficit-score (n = 10/group). As standard practice^20^, healthy animals had to pass the test with 0 or 1 point to be included in the study. No animal had to be excluded for this reason. In accordance with moderate brain trauma, the results indicated functional deficits in all CCI animals. Animals treated with DNase-I showed at 1 dpi no difference compared to Vehicle (Fig. 1c). Motor function was tested with Rotarod at 5 dpi and showed no differences between the groups (n = 12/group, Fig. 1d). The course of body weight and neuro-deficit-scores up to 5 dpi were not different between treatment groups (Supplementary Information 1).

### Influence of DNase-I treatment on brain edema formation and BBB integrity after CCI

**Figure 2:**
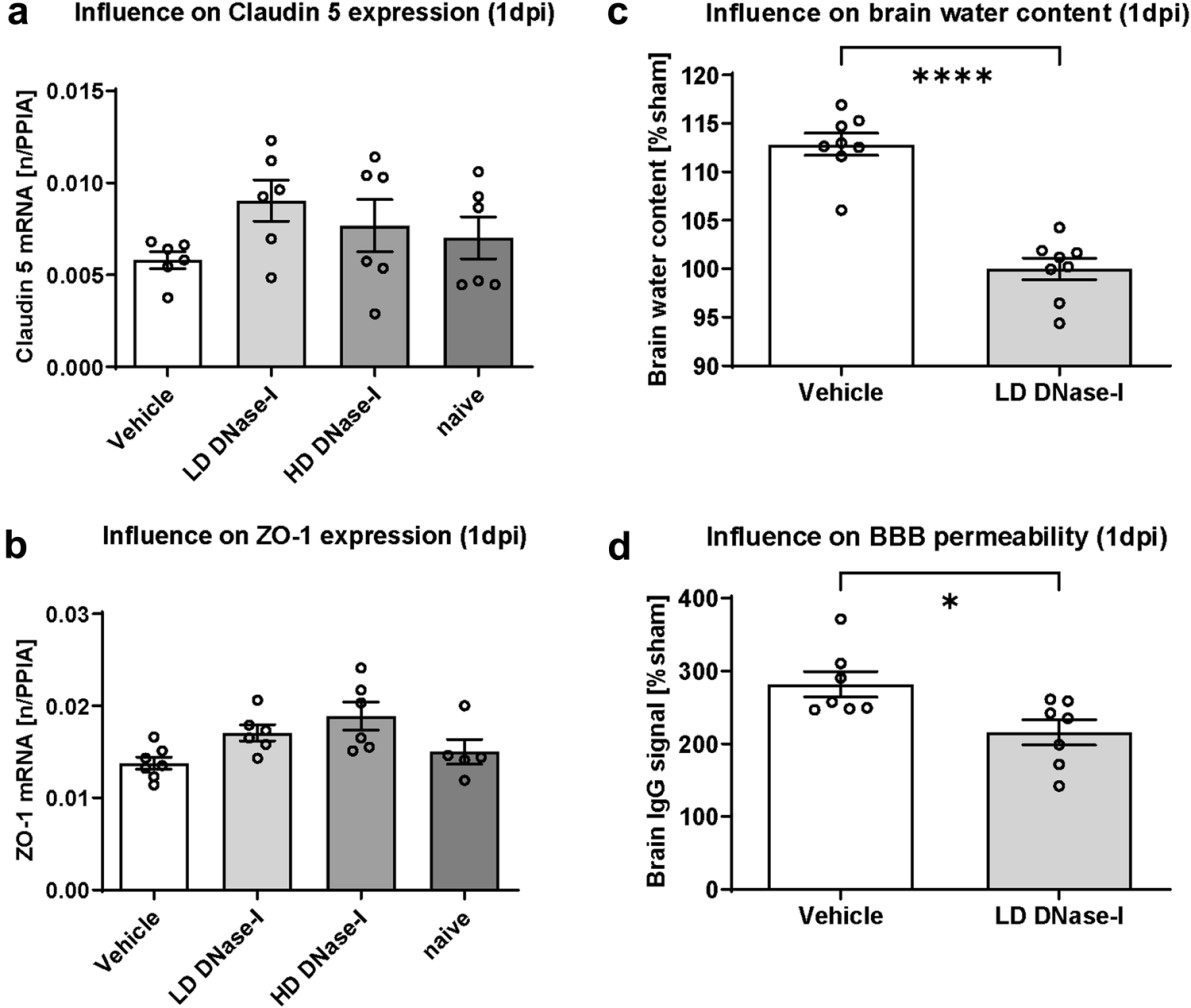
Regulation of cerebral edema severity and blood–brain barrier (BBB) integrity post-CCI. (**a b**) Upregulation of the mRNA levels of tight junction proteins *Claudin-5* (**a**) and *ZO-1* (**b**) following DNase-I treatment (n = 10 mice/group), consistent with BBB preservation. (**c**) Brain water 24 h post-CCI (1 dpi) was significantly lower in the LD DNase-I (2 mg/kg) group (n = 8 mice/group). Brain water content and IgG levels were determined in a separate set of animals with the best effective dosage in terms of lesion volume (2A): (**d**) BBB integrity 1 dpi as evidenced by immunoglobulin G (IgG) extravasation. Blots were revealed by near infrared laser scanning, quantification was performed with ImageJ. Levels were significantly lower in DNase-I-treated animals (n = 8 mice/group). A significance level p < 0.05 is marked with *, p < 0.01 with **, and p < 0.001 with ***. All data are presented as mean ± SEM; *p* values are adjusted for multiple comparisons by Sidak correction.

One of the leading factors contributing to secondary brain damage is the formation of cerebral edema. To maintain BBB integrity, adequate structural support from the tight-junction (TJ) proteins is essential^21,22^. We therefore quantified gene expression in injured hemispheres at 1 dpi using qPCR (n = 10 per group). Expression of *Claudin-5* and *ZO-1* mRNA levels was not significantly higher after treatment with LD-DNase-I (Fig. 2a,b).

In a next step, mice were randomized to CCI with LD-DNase-I (most effective dose regarding lesion) or vehicle treatment to examine edema formation after TBI (n = 10 per group). Brain water content was determined by wet/dry ratio 24 h after CCI. Treatment with DNase-I reduced cerebral edema significantly (Fig. 2c, results as mean ± SEM: Vehicle 112.8 ± 1.15%Sham, LD-DNase-I 100 ± 1.12% sham, Vehicle vs. LD-DNase-I *p* < 0.0001; n = 8/group). Brain edema may be cytotoxic or vasogenic in character. To determine if DNase-I treatment shrinks cytotoxic edema or stabilizes the BBB, IgG extravasation was quantified as a surrogate parameter for BBB permeability after CCI. IgG brain content was quantified by anti-IgG dot blotting^23^. LD DNase-I sufficiently reduced IgG extravasation up to 67% compared to vehicle solution (Fig. 2d and supplementary figure, results as mean ± SEM: Vehicle 282.1 ± 17.56% sham, LD-DNase-I 215.7 ± 17.33%Sham, Vehicle vs. LD-DNase-I *p* = 0.019; n = 8/group). The data indicate that DNase-I treatment reduces posttraumatic brain edema and BBB permeability after CCI in mice.

### Influence of DNase-I treatment on cerebral inflammation

**Figure 3:**
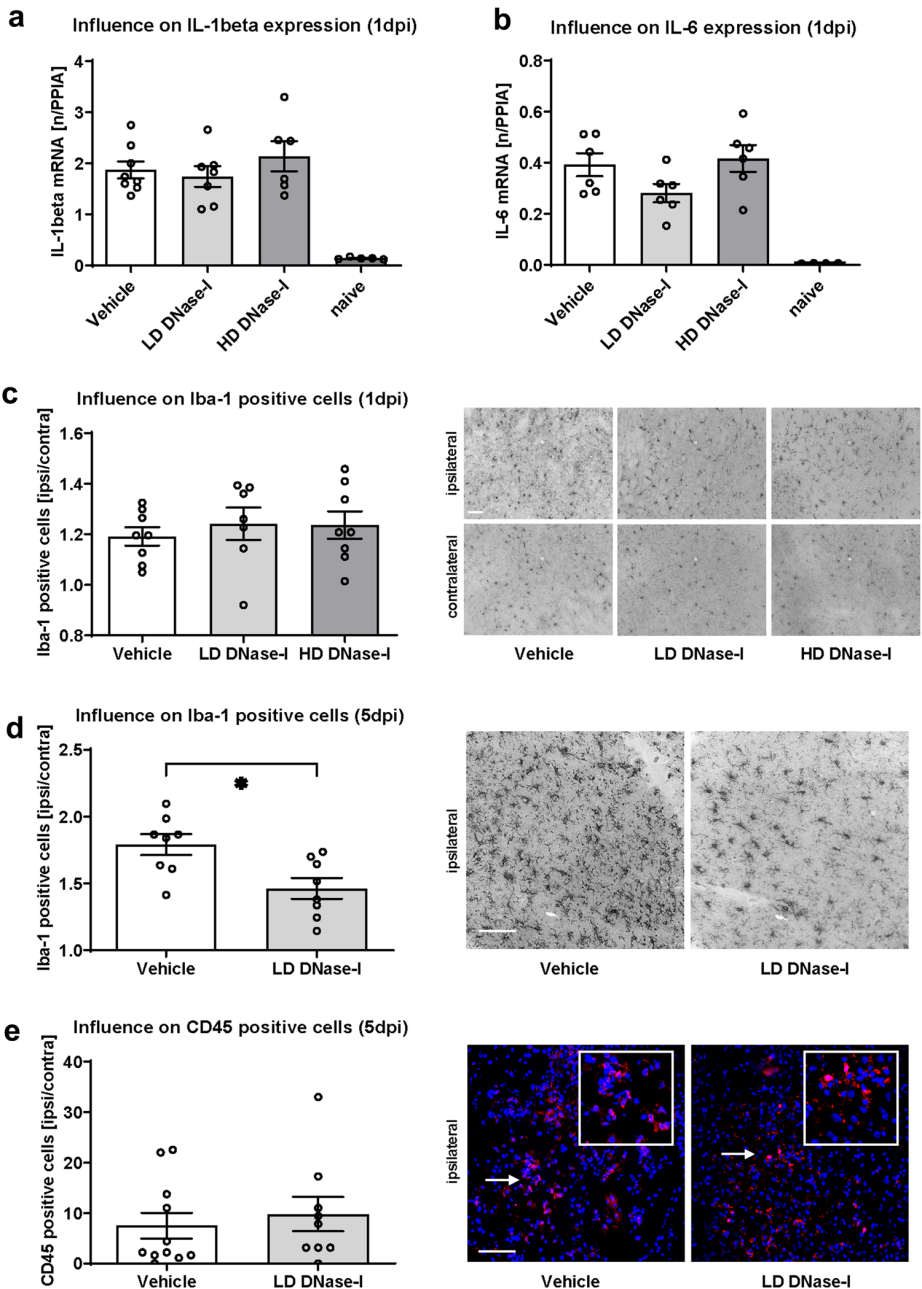
Effects of DNase-I injection on inflammation markers after traumatic brain injury. (**a**,**b**) Effects of DNase-I on pro-inflammatory marker genes *IL-1* $\beta$ (**a**) and *IL-6* (**b**) as measured by qPCR at 24-h post-controlled-cortical-impact (CCI, 1 dpi). Scale bars 50 µm each in all images 4C, 4D and 4E. (**c**) Effects of DNase-I on the number of activated perilesional microglia as evidenced by Iba-1 immunostaining. DNase-I did not substantially reduce neuroinflammation at 1 dpi (n = 10 mice/group). (**d**) The pericontusional increase in Iba1 + cells was significant lower in LD DNase-I treated mice than vehicle-treated mice at 120 h post-CCI (5 dpi) but (**e**) treatment did not alter the number of CD45 + cells (n = 12 mice/group). A significance level p < 0.05 is marked with *, p < 0.01 with **, and p < 0.001 with ***. All data are presented as mean ± SEM; *p* values are adjusted for multiple comparisons by Sidak correction.

In parallel with the development of cerebral edema, an overshooting immune response is progressing, which also contributes to secondary brain damage after TBI. To determine if cerebral inflammation is also modulated by DNase-I treatment, mRNA expression of *IL-1* $beta$ (Fig. 3a) and *IL-6* (Fig. 3b) was examined 24 h after CCI (1 dpi). Cytokine mRNA expression of both genes did not differ between the groups.

In addition, activated microglia cells were quantified by immunohistochemistry using antibodies specific to Iba-1. The number of Iba-1 positive cells increased in the lesioned hemisphere compared to contralateral at 1 dpi without differences between treatment groups (Fig. 3c). At 5 dpi, a significant difference was evident between the different treatments and less Iba1 positive cells were found in lesioned hemispheres of LD-DNAse1-I-treated animals (Fig. 3d, results as mean ± SEM: Vehicle 1.79 ± 0.08 fold change ipsi/contra, LD-DNase-I 1.46 ± 0.08 fold change ipsi/contra, Vehicle vs. LD-DNase-I *p* = 0.0104; n = 12/group). However, immunohistochemistry using anti-CD45^24^, demonstrated more CD45 positive cells in the ipsilateral compared to the contralateral hemisphere, but without differences between treatment and control groups at 5 dpi (Fig. 3e).

### Influence of DNase-I treatment on clot formation and cerebral perfusion

**Figure 4:**
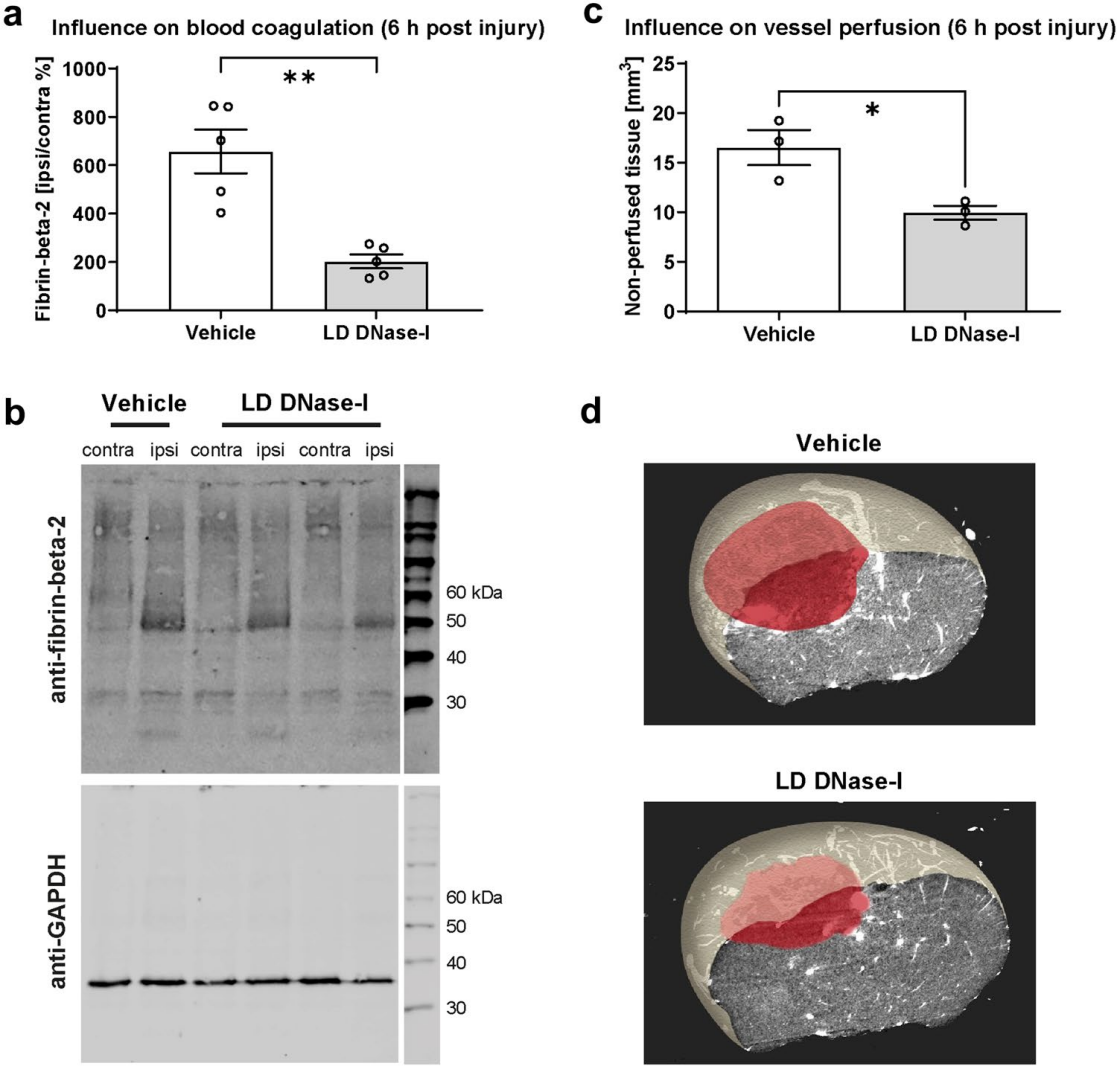
Regulation of coagulation and vessel perfusion post-CCI. (**a**,**b**) Western blotting of Fibrin-Beta-2 6 h post-CCI, blots were revealed by near infrared laser scanning, quantification was performed with ImageJ, indicating decreased expression following LD DNase-I treatment compared to vehicle (n = 5 mice/group). (**c**,**d**) Characterization of thrombus formation using cast material injection and micro-CT imaging (n = 3 mice/group). The area of non-perfused brain tissue (red shading) did not differ significantly between treatment groups. A significance level p < 0.05 is marked with *, p < 0.01 with **, and p < 0.001 with ***. All data are presented as mean ± SEM; *p* values are adjusted for multiple comparisons by Sidak correction.

We next examined the influence of DNAse-I treatment on posttraumatic clot formation in two different ways: (a) the brain tissue of the animals was examined with an antibody specific for clotted fibrin. The difference between DNase-I and vehicle treatment was determined 6 h after CCI. This time point was previously shown to display major changes in the coagulation system^25^. The amount of clotted fibrin was decreased 6 h after CCI in LD DNase-I treated animals compared to Vehicle (Fig. 4a,b, results as mean ± SEM: Vehicle 657.3 ± 90.32 fibrin-beta-2 ipsi/contra %, LD-DNase-I 202.5 ± 28.55 Fibrin-Beta-2 ipsi/contra %, Vehicle vs. LD-DNase-I *p* = 0.0014; n = 5/group). (b) Vascular exploration using micro-computer-tomography showed a significant smaller volume of the non-perfused area in LD DNase-I treated animals, which could lead to better perfusion (Fig. 4c,d, results as mean ± SEM: Vehicle 13.17 ± 1.78 mm^3^, LD-DNase-I 9.95 ± 1.23 mm^3^, Vehicle vs. LD DNase-I *p* = 0.027; n = 3/group).

## Discussion

This study demonstrates a neuroprotective effect of DNase-I treatment in the early phase 1 day after TBI (1 dpi) with delayed lesion growth and limited vasogenic cerebral edema. Even 5 days after trauma (5 dpi), microglial activity was persistently reduced in the injured hemisphere. These effects indicate a relevant role of cfDNA in the development of secondary brain damage after TBI.

cfDNA can be both, a diagnostic parameter^5,9,26,27^ and a therapeutic target after TBI. DNase-I is fairly affordable, and even already being used in the treatment of cystic fibrosis in humans, where extracellular chromatin is generated in the lung^28^. Here, we show that DNase-I treatment has also protective effects after TBI in mice. DNase-I delayed lesion growth from 1 to 5 dpi, which could generate time to stabilize the coagulation system before surgical intervention like decompressive craniectomy or transport of the patient to a specialized medical center with optimal facilities.

Sustained inflammation and microglial activation continue for months post-injury both in patients and in the CCI model of TBI^29,30^ and are responsible for neuronal damage and CNS dysfunction after TBI^31^. We report significantly reduced numbers of Iba1 positive cells of DNase-I treated mice at 5 dpi. On the other hand, we did not observe differences in *IL-1beta* and *IL-6* mRNA expression at 1 dpi and did not found any difference in the numbers of CD45 positive cells at 5 dpi. However, previous studies suggested that microglia-related effects may relate to Toll-like-Receptor (TLR) activation via cfDNA: inhibition of the TLR4 signaling pathway in intracerebral hemorrhage in mice reduced the amount of M1-like microglial polarization and improved early functional outcomes^32^. Detection of TLR2 and TLR4 in blood samples from stroke patients are independently associated with poor outcome increased lesion volume after stroke^33^ and TLR4 worsens outcome after stroke in mice^34^. The anti-inflammatory effect at 5 dpi after controlled cortical impact (CCI) may be partly due to indirect inhibition of TLR activation via degradation of cfDNA by DNase-I. This pathway will be the target of further investigation.

DNase I treatment contributes also to the reduction of vasogenic cerebral edema after CCI, as indicated by the reduction in brain water content and IgG extravasation in our study. The underlying mechanism needs to be elucidated in further studies.

A possible explanation for the observation of the neuroprotective effect of DNase-I could be the elimination, respectively cleavage of chromatin material which acts as a procoagulant factor: NETs interrupt the cerebral circulation and are approved thrombus components in cerebral ischemia^35,36^. Lysis of these NET-thrombi with DNase-I has already been successfully performed after cerebral ischemia in mice^19^. cfDNA itself has a prothrombotic and procoagulant effect^37,38^. With significant diminished fibrin cleavage products and significantly reduced non-perfused brain volume 6 h after CCI, our study provides several indications that DNase-I could also be involved in lesion development after TBI. It was shown before that blood-coagulation capacity of cfDNA correlates with polymer size and structure^39^, short polymers can even act as competitors and reduce blood coagulation triggering of large fragments of cfDNA^40^. DNase-I could reduce the procoagulant state after TBI^41^ by cleaving long DNA polymers into short parts, which are less coagulant. Blood aggregation is also activated by reduced blood flow due to brain edema, via the stressed endothelium and the overshooting immune response. DNase-I could have an impact on all three pathways. Activated neutrophils extrude neutrophil extracellular traps that not only ligate pathogens and cfDNA, but also provide an intense stimulus for clot formation^38,42^. Von Willebrand factor increases in plasma following severe TBI and could be a marker of unfavorable outcome^43^. Von Willebrand factor interacts with extracellular DNA traps and plays a crucial role in stroke^44^. Whether cfDNA binds to von Willebrand factor and exaggerates secondary brain damage after TBI remains to be established. On the other hand, TBI may cause systemic coagulopathy^45^ in addition to, or in contrast to, local perfusion deficits due to thrombus formation^12^. In this case and in principle, systemic administration of DNase I could support the initiation of coagulopathy by the mechanisms described above—with the known harmful consequences. Whether and in which direction cfDNA or DNase-I influence the coagulation system after TBI are promising targets for further studies.

Further studies are now required to elucidate the exact underlying mechanisms of cfDNA induced brain injury and to establish whether chromatin release could also contribute to the cognitive decline after TBI. It might be possible that DNase I therapy represents a cost-effective, easy-to-use supportive treatment strategy for TBI.

Despite clear data on brain edema formation and early histological brain damage, the present study fails to provide evidence for functional improvement by DNase-1 therapy after experimental TBI. Neuro-deficit-score and Rotarod data failed to show any effects at 1 dpi and 5 dpi. Reduction in extent of brain edema, blood–brain-barrier disruption and brain lesion at 1 dpi therefore does not result in improved functional recovery. At 5 dpi, brain lesion volume was similar between groups, suggesting that the initial putative beneficial effect was not long lasting and not sufficient to result in functional improvement.

The effect on blood coagulation and vascular tree was investigated in very few animals and was not the main objective of this study. A more detailed examination of the influence of cfDNA on blood coagulation after TBI with appropriate laboratory analyses, immunohistochemical staining and perfusion studies by MRI should be performed in a separate study.

To decrease the number of animals, this study mainly focused on the effect of DNase-I in brain-injured animals. Therefore, the influx of DNase-I in sham operated or healthy animals was not studied in detail. Only in experiments to determine the brain water content the DNase-I was administered to sham animals. In these animals, neurological function and body weight development was not adversely affected by DNase-I. This suggests that there are no acute significant side effects due to DNase I treatment. Furthermore, this is a substance already approved for the treatment of humans, however for a different indication. In future studies, the effect in sham or naïve animals should be investigated in more detail to rule out any negative effect of DNase-I.

In addition, only male animals are used in the study; this is to minimize the number of animals, as cycle-dependent hormonal fluctuations in female animals could increase the standard deviation^46,47^. We therefore cannot estimate the gender effect and the impact of DNase-I treatment in braininjured female mice. These effects need to be addressed in future studies.

In summary, DNase-I treatment in the acute phase after TBI delays lesion growth, diminishes vasogenic brain edema and improves perilesional perfusion in the early phase after TBI. At 5 dpi, the number of microglial cells was reduced. The persistent effect with respect to microglia 5 dpi may be due to the early influence on the coagulation system and the reduction of brain edema by DNase-I treatment after CCI in mice.

## Methods

### Animals and experimental protocol

The study was approved by the German animal protection legislation (G-13-1-074, State Inspection Office Rhineland-Palatinate, Germany). In accordance to the ARRIVE Guidelines^48^ before and during experiments, animals were housed in compliance with institutional guidelines of the Johannes Gutenberg-University, Mainz. The study was conducted in 84 male C57BL/6N mice (Charles River Laboratories, Sulzfeld, Germany; 22–28 g). The following exclusion criteria were applied: Severe behavioral disturbances (stupor, decreased locomotion, convulsions); impactor dysfunction, and severe body weight loss (> 15%) associated with pain symptoms (teeth grinding). Overall, 8 of 84 animals met the exclusion criteria and were euthanized. The total number of animals was minimized by continuing the evaluation of further effects concerning blood–brain-barrier (BBB), coagulation and perfusion as well as the experiments with a survival time of 5 days after lesion (5 dpi) only with the more effective low dose (LD) from the first series of experiments with 1 dpi survival time.

**Figure 5:**
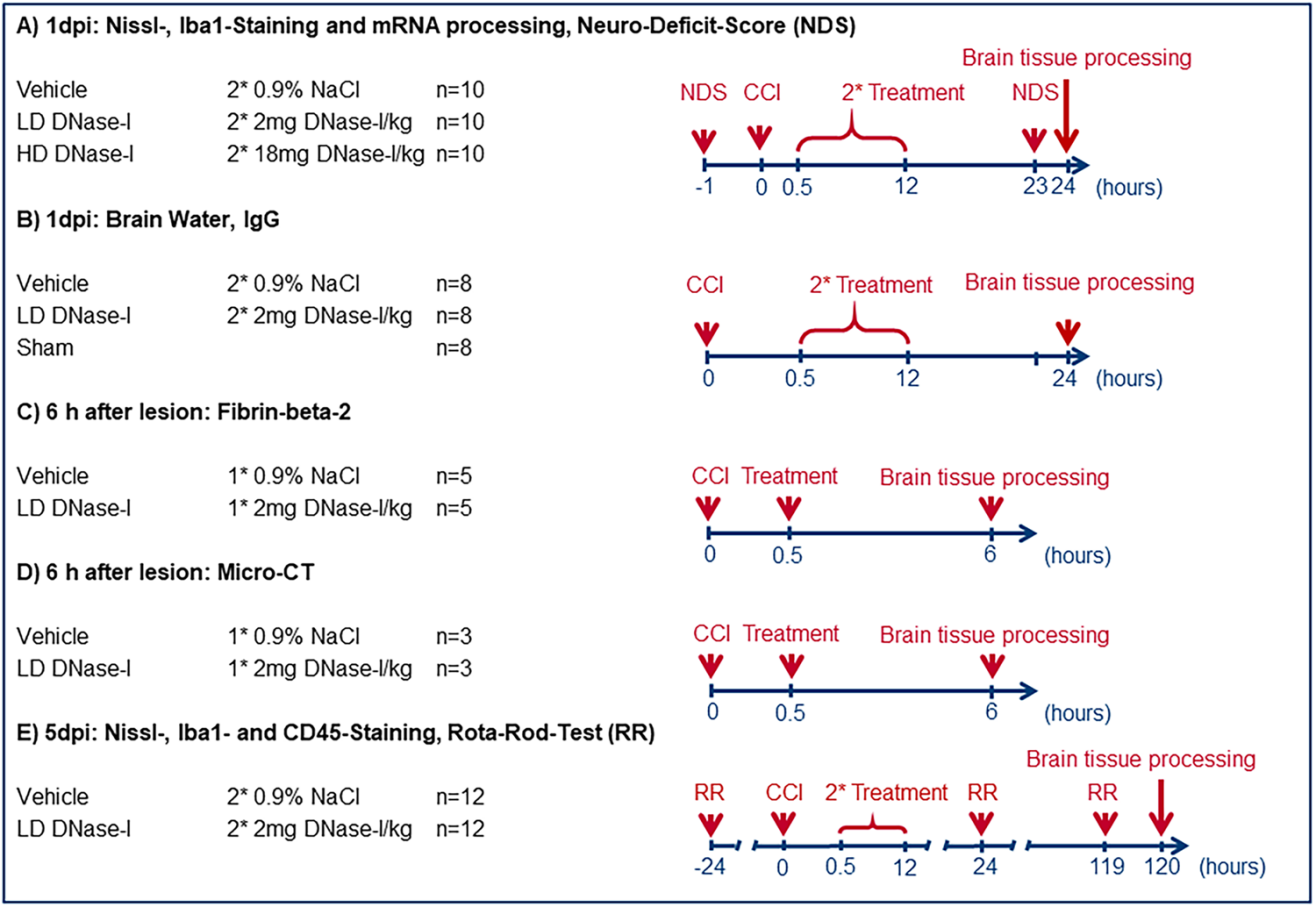
Study design. Overview of the different experiments performed separately with the respective dosage of DNase-I and number of animals. First, lesion volume 1 day post injury (1 dpi) was determined after using different doses of DNase-I and vehicle. Brain tissue from the same cohort was used to perform qPCR. The experiments in sections B, C, D, and E were continued only with the effective low dose of DNase-I (LD DNase-I vs. vehicle and/or sham).

The study is a prospective, randomized, triple-blinded in vivo study. A randomized blinding list was established. During the experiments and the evaluation, the group assignment was known only to the person who dosed the syringes with the drugs or the vehicle solution. This person was not otherwise involved in the conduction of the study. The syringe contents were taped off and were not later identifiable, labeled only with an experimental animal number. All individuals who participated in the CCI/sham procedure, neurological as well as motor testing, tissue preparation, histological as well as immunohistological staining, mRNA/protein detection, micro-CT examination and analysis were blinded throughout the experimental procedure. Unblinding occurred during the statistical analysis. A graphical overview of the study design is attached (Fig. 5).

To test the therapeutic value of DNase-I after TBI, animals were randomly assigned to receive low- (LD: 2 mg/kg, n = 10/group), high-dose treatment (HD: 18 mg/kg, n = 10/group) or vehicle-solution (2 × 500 μL NaCl 0.9%, n = 10/group), injected intraperitoneally (i.p.) 30 min and repeated 12 h after brain injury. The applied dosages are in accordance with the dosages used in similar experiments with rodents^49^. At 1 dpi neurologic function, lesion-volume, Iba1 activation, IgG-extravasation and qPCR of tight junction and inflammatory markers were examined.

Afterwards DNase-I LD (better result than DNase-I HD in first part of experiment) was compared to Vehicle and sham operated animals, regarding brain edema formation 1 dpi (n = 8/group). In previous experiments we could demonstrate the maximum influence of TBI on blood coagulation 6 h after trauma^25^, therefore this interval was chosen for investigations by using micro-CT for vessel obstruction CCI (n = 3/group) and Wester blotting for cleavage of fibrin (fibrin-beta-2) 6 h after trauma (n = 5/group).

After a prolonged survival of 5 dpi, the motor outcome was determined, lesion volume and the immigration and activation of immune cells by IHC was investigated (DNase-I LD vs. Vehicle; n = 12/group).

After intraperitoneal anesthesia, as described above, brains were removed in deep anesthesia immediately after decapitation, or transcardiac perfusion was performed in preparation for micro-CT examination.

### Drug preparation

Deoxyribonuclease-I (DNase-I, Pulmozyme^®^, dornase alfa, Roche Grenzach-Wyhlen, Germany) was dissolved in 0.1 ml 0.9% sodium chloride solution. Doses of low dose 2 mg/kg DNase-I (LD DNase-I), high dose 18 mg/kg DNase-I (HD DNase-I) or vehicle solution were given intraperitoneally to animals 30 min after insult and was repeated 12 h after insult. The doses used are based on previous studies, in which the quantities did not cause any damage to the animals and where detectable effects of DNase-I were observed in similar pathologies^12,19,50–54^.

### Controlled cortical impact

Traumatic brain injury was induced with an electromagnetic impact device as controlled cortical impact (CCI, Leica Impact One™ Stereotaxic Impactor, Richmond, IL; tip diameter: 3 mm; impact velocity: 6 m/s; impact duration: 200 ms; displacement: 1.5 mm)^55^. Animals were anaesthetized with a triple combination of 5 mg/kg Midazolam (Ratiopharm, Ulm, Germany), 0.05 mg/kg Fentanyl (Curamed, Karlsruhe, Germany) 0.5 mg/kg Medetomidine Pfizer, Karlsruhe Germany) intraperitoneally. Rectal temperature was maintained and controlled at 37 °C during anesthesia. After craniotomy, trauma was induced on the right parietal cortex, center bregma − 2 mm, as defined by the Mouse Brain Library-Atlas (http://www.mbl.org). Subsequently the craniotomy was closed with the original boneflap and fixed with tissue adhesive (Histoacryl, Braun-Melsungen, Melsungen, Germany), anesthesia antagonized with 0.5 mg/kg Flumazenil (Anexate^®^, Roche AG, Basel Switzerland) and 2.5 mg/kg Atepamezolhydrochlorid (Antisedan^®^, Vetoquinol AG, Bern, Switzerland), afterwards the animals were transferred into their cages, which were placed for 1.5 h in a neonatal incubator (IC8000, Draeger, Luebeck, Germany) with controlled air temperature (35 °C) and ambient humidity (35%)^56,57^. The experimenter who carried out the CCI procedure was blinded to the treatment. A separate experimenter, also blinded to treatment and CCI or Sham procedure, performed the behavioral tests, tissue preparation, and histological analysis.

### Functional outcome

Before CCI and after injury an investigator blinded to the experimental groups determined the functional outcome with a neuro-deficit-score ranging from 0 (healthy, successful in all tasks) to 15 (severely impaired) points (n = 30), sham animals achieve only 0–2 points in our standard experimental setup^58^. We adapted a previously established score for the present study^59^: the neuro-deficit-score consists of 10 different tasks evaluating motor ability, alertness, balancing, and general behavior. One to two points were awarded for failure to successfully perform a task. For survival up to 5 dpi (n = 24), rotarod training was completed 3 days before CCI, and a rotarod motor test (Rotarod Treadmill, MED Associates, INC, St Albans, VT) was performed 1 day before CCI, 1 day after (1 dpi) and 5 days after (5 dpi) CCI. An accelerating rotarod test was performed, with the rotarod speed increasing linearly from 4 to 40 rpm over 5 min. The test was terminated when the mice fell from the bars. Each mouse was placed on an accelerating rotating cylinder, and the time from the start of the rotation until the animal fell (27 cm drop height) was automatically recorded. One day before CCI, on the first and fifth days after trauma, two trials were performed in direct succession, followed by a 10-min rest and then again two trials in direct succession. Three days before CCI, two complete runs were performed as exercises, without time recording. The longest documented running time of a mouse before CCI was 102 s. The mean value of each animal 1 day before CCI was defined as 100% "baseline". At 1 dpi and 5 dpi, animals were retested in two trials per time point. The results of the postinjury trials were averaged and evaluated relative to the preinjury latencies to account for the deficit in motor performance^56^. As our group had previously shown, rotarod performance of sham-injured animals between 24 and 168 h after CCI did not differ from the performance of animals without any intervention^55^. Therefore, in accordance with ARRIVE guidelines, no sham group was used in this arm of the experimental design to minimize the number of experimental animals.

### Nissl-, Iba1- and CD45-staining

The animals were euthanized in deep anesthesia, brains were removed immediately after decapitation, frozen in powdered dry ice for 5 min and stored at − 30 °C until histological processing (1 dpi: n = 10/group, 5 dpi: n = 12/group)^56^. The animals were not perfused transcardially. 10-μm-thick coronal sections were cut at 500-μm intervals throughout the brain, placed on Superfrost plus slides (Thermo Fisher Scientific, Germany) and stained with cresyl violet or further processed by immunohistochemistry. The first sample collected at Bregma + 3.14 mm, as defined by the Mouse Brain Library-Atlas (http://www.mbl.org). With a computerized image system (DeltaPix InSight, Smorum, Denmark) the cresyl violet stained sections were analyzed to determine brain lesion volume.

The remaining ipsilateral area marked in the Nissl stain was subtracted from the contralateral hemisphere, to minimize distortion due to edema, the difference was considered the lesion area.

Lesion volumes were calculated by multiplying lesion areas obtained from 16 consecutive sections with the distance interval of 500 µm (0.5 × (A 1 + A 2 + A 3 + ⋯ + A 16))^58^. For anti-Iba1 immunohistochemistry, sections were fixed in 4% paraformaldehyde in phosphate buffered saline and incubated with blocking serum (5% Normal Goat Serum, Biorad, CA, USA; 2% BSA GE Europe, Germany) at RT^4^. Immunohistochemistry was performed with anti-Iba-1 (rabbit, 1:1500, Wako Pure Chemical Industries, Osaka, Japan). Secondary antibody was biotinylated anti-rabbit IgG (Vector Laboratories Inc., Burlingame, CA, USA). Signals were detected using ABC Complex (Vector) and DAB (Thermofischer, Waltham, MA, USA). The total number of positive cells was quantified in two sections (bregma − 1.82 mm, www.mbl.org). Images were acquired at 20× magnification and cells were counted in pericontusional and contralateral regions in 2 serial sections (ROI: 2.55 mm^2^). In the same area, the CD45 positive cells (1:500, rat anti CD45, Thermo-Fisher, Waltham, MA) were counted at 5 dpi. CD45 staining followed by goat anti-rat IgG (1:500, Alexa Fluor 568, Thermo-Fisher, Waltham, MA) and DAPI as a nucleus counterstain was performed (1:10,000, Thermo-Fisher, Waltham, MA). Photographed under 20× magnification, cells were counted in the perilesional and corresponding non-injured contralateral regions of two serial sections with the largest average lesion area (ROI: 2.55 mm^2^, bregma − 1.64 mm and bregma − 1.82 mm, www.mbl.org, respectively). Throughout the area, immunolabeled cells were counted by an investigator blinded to treatment using software-assisted counting (ImageJ, Java open-source software, NIH)^60^.

### RNA isolation and quantitative polymerase chain reaction

During histological processing, samples (n = 10/group) were collected from the injured brain region, frozen in liquid nitrogen, and stored at − 80 °C^61^. RNA extraction, reverse transcription and mRNA quantification by real-time quantitative polymerase chain reaction (qPCR) was performed as described^62^ using established primers and probes^24^. Sequences of applied primer pairs (50–30): PPIA: GCGTCTSCTTCGAGCTGTT; *IL-1beta*: GTGCTGTCGGACCCATATGAG; *IL-6: GAGGATACCACTCCCAACAGACC;* Claudin-5: CGTTGGAAATTCTGGGTCTG; ZO-1: CTCAACACACCACCATTgCTgTT. A standard curve for absolute quantification was generated for each PCR product as previously described^63^ and the absolute copy numbers of the target genes were normalized to the absolute copy numbers of the reference gene cyclophilin A (PPIA).

### Brain water content

Animals were sacrificed 1 dpi under deep anesthesia as describe above (n = 30). Cerebellum was separated and the hemispheres were cut along the interhemispheric plane and each hemisphere centered by the lesion into two 3 mm thick sections. One quadrant of each hemisphere was dried in a vacuum-centrifuge (Univapo 100 H, UniEquip, Planegg, Germany) for 48 h. Based on the gravimetrical differences, brain water content was obtained by the following calculation: water content (%): (WW − DW)/WW × 100, where WW is the wet weight (g) and DW, the dry weight (g) of the sample^64^.

### Quantification of brain IgG and fibrin fragments

Brain samples were lysed in ice-cold lysis buffer (Thermo Fisher Scientific) containing protease inhibitor cocktail (Roche). For dot-blot experiments, equal amounts of protein (16 µg) were spotted on a nitrocellulose membrane and IgG was revealed using IRDye 800 goat anti-mouse IgG antibody (n = 30, #926-32210, LI-COR, Lincoln, Nebraska, USA; n = 8/group). For western blot experiments, a specific antibody directed to clotted fibrin-beta-2 (BSS 15-42, Gentaur) was used in a separate set of animals (n = 6/group). In a previous study it became apparent that differences in the activation of the coagulation system are most likely to be present 6 h after TBI^24^. For this reason, a separate cohort (n = 6/group) was formed with 6 h survival for fibrin quantification^25^. For reference antibody against GAPDH (Acris, clone 6C5) was applied. Blots and their optical signal intensities were revealed by near infrared laser scanning using the LI-COR Odyssey imaging system. Quantification was performed with ImageJ (NIH, MD, USA)^56^.

### Brain vessel imaging by micro-computer tomography

After intraperitoneal anesthesia like described above, animals (n = 3/group) were transcardially perfused. Perfusion was performed with a cannula in the left ventricle (21G, B Braun Melsungen AG, Melsungen, Germany) and opened right atrium. After perfusion with PBS (Sigma Aldrich, Hamburg, Germany) and Heparin (B Braun) 4% paraformaldehyde (Sigma-Aldrich) was followed, all under controlled temperature and perfusion pressure (n = 6). After perfusion with PFA, both the descending aorta and the inferior vena cava were clamped and transcardial perfusion was continued for 20 min with Microfil^®^ MV-122 (Flowtech Inc., Carver, MA, USA) at a constant rate of 0.2 ml/min. Subsequently, the skulls were decalcified by incubating these in 8% formic acid (Sigma-Aldrich) at room temperature for 48 h. The skull was then transferred to a 4% PFA solution stored at 4 °C ^65^. An industrial micro-CT system (μCT40, Scanco Medical AG, Brüttisellen, Switzerland) was used to obtain Dicom datasets of the murine brains (voxel size of 20 µm). These data were imported into Amira^®^ software version 5.4.2 (FEI Visualization Sciences Group, Hillsboro, OR, USA). To assess the size of the cerebral lesions, the devascularized area was identified and marked in coronary sections using the SegmentationEditor by a blinded investigator and the volumes of the lesions were subsequently calculated^66^. These are final experiments and the processing is very complex. Micro-CT examination of perfusion is a new method for creating a skeleton of the entire vascular bed, including arteries, veins, and capillaries.

### Statistics

All experiments were performed and analyzed in a randomized (computer-based randomization) and triple-blinded manner. Statistical analysis was performed with GraphPad Prism 9 statistical software (GraphPad Software Inc., La Jolla, CA, USA). To determine the optimal sample size, an a priori power analysis using G ∗ Power^67^ was performed using lesion volume data from previously published studies^24,56,60,68^. The a priori power analysis for the effect size of 0.7 suggests that a standard statistical power (1 − β) of Pβ = 0.95 for a given significance level (α) of 0.05 can be obtained for lesion volume with 10 subjects per group (3 groups) and brain water content with 10 subjects per group. For 5 days of survival, the group size was expanded (n = 12/group). Prior to statistical analysis, we checked the test assumptions. Due to the limited power in small samples, we did not perform formal goodness-of-fit tests prior to the t test or ANOVA, but instead relied on the graphical assessment of distribution characteristics^69^. Normality was checked by using normal probability Q–Q plots and inspecting the unimodality and symmetry of histograms. The equality of variances was checked by inspecting histograms and standard deviations. For comparison of multiple independent groups, Brown-Forsythe and Welch ANOVA with post-hoc Dunnett T3 multiple comparisons test (comparisons between all groups) was employed. To evaluate group differences in repeated-measurements from the same animals (rotarod), RM two-way ANOVA was applied (factors: treatment and time), followed by Šidáks multiple comparisons test. Comparisons between two independent groups were carried out by the Welch’s t test. Values of *p* < 0.05 were considered significant. A significance level p < 0.05 is marked with *, p < 0.01 with **, and p < 0.001 with *** in the figures. Data are presented as the mean and standard error of means (mean ± SEM).

### Ethical approval

The study was approved by the German animal protection legislation (G-13-1-074, State Inspection Office Rhineland-Palatinate, Germany). In accordance to the ARRIVE Guidelines^48^ before and during experiments, animals were housed in compliance with institutional guidelines of the Johannes Gutenberg-University, Mainz, Germany. The total number of animals was minimized by carrying out further experiments with only one dose of the drug and omitting sham groups if possible. All methods were carried out in accordance with relevant guidelines and regulations. Results and methods are also reported according to the Arrive Guidelines.

## Supplementary Information

Supplementary Information.

## Data Availability

The data that support the findings of this study are available from the author with overall responsibility, ST, upon reasonable request.
