# Supplementary material for: Early DNase-I therapy delays secondary brain damage after traumatic brain injury in adult mice
Source: Sci Rep. 2023 Mar 16;13:4348. doi: 10.1038/s41598-023-30421-5 (PMC10018640; doi:10.1038/s41598-023-30421-5)
Supplement: Supplementary file 1 — Supplementary Information. [file 41598_2023_30421_MOESM1_ESM.pdf]

Supplementary Information

Early DNase-I therapy delays secondary brain damage after traumatic brain injury in adult mice

Tobias J. Krämer[1,2], Florian Pickart[1], Bruno Pöttker[1], Christina Gölz[1], Axel Neulen[3], Tobias Pantel[3], Hermann Goetz[4], Katharina Ritter[1,] Michael K. E. Schäfer[1, 5, 6, 7*] & Serge C. Thal[1, 5, 7, 8*]

[1]Department of Anesthesiology, University Medical Center of Johannes Gutenberg University, Langenbeckstrasse 1, 55131 Mainz, Germany.

[2]Faculty of Health, University Witten/Herdecke, Witten, Germany.

[3]Department of Neurosurgery, University Medical Center of Johannes Gutenberg University, Langenbeckstrasse 1, 55131 Mainz, Germany.

[4]Cell Biology Unit, University Medical Center of Johannes Gutenberg University, Langenbeckstrasse 1, 55131 Mainz, Germany.

[5]Focus Program Translational Neurosciences, University Medical Center of Johannes Gutenberg University, Langenbeckstrasse 1, 55131 Mainz, Germany.

[6]Research Center for Immunotherapy, University Medical Center of Johannes Gutenberg University, Langenbeckstrasse 1, 55131 Mainz, Germany.

[7]Center for Molecular Surgical Research, University Medical Center of Johannes Gutenberg University, Langenbeckstrasse 1, 55131 Mainz, Germany.

[8]Department of Anesthesiology, Helios University Hospital Wuppertal, University Witten/Herdecke, Heusnerstrasse 40, 42283 Wuppertal, Germany.

* equal contribution

Figure S1:

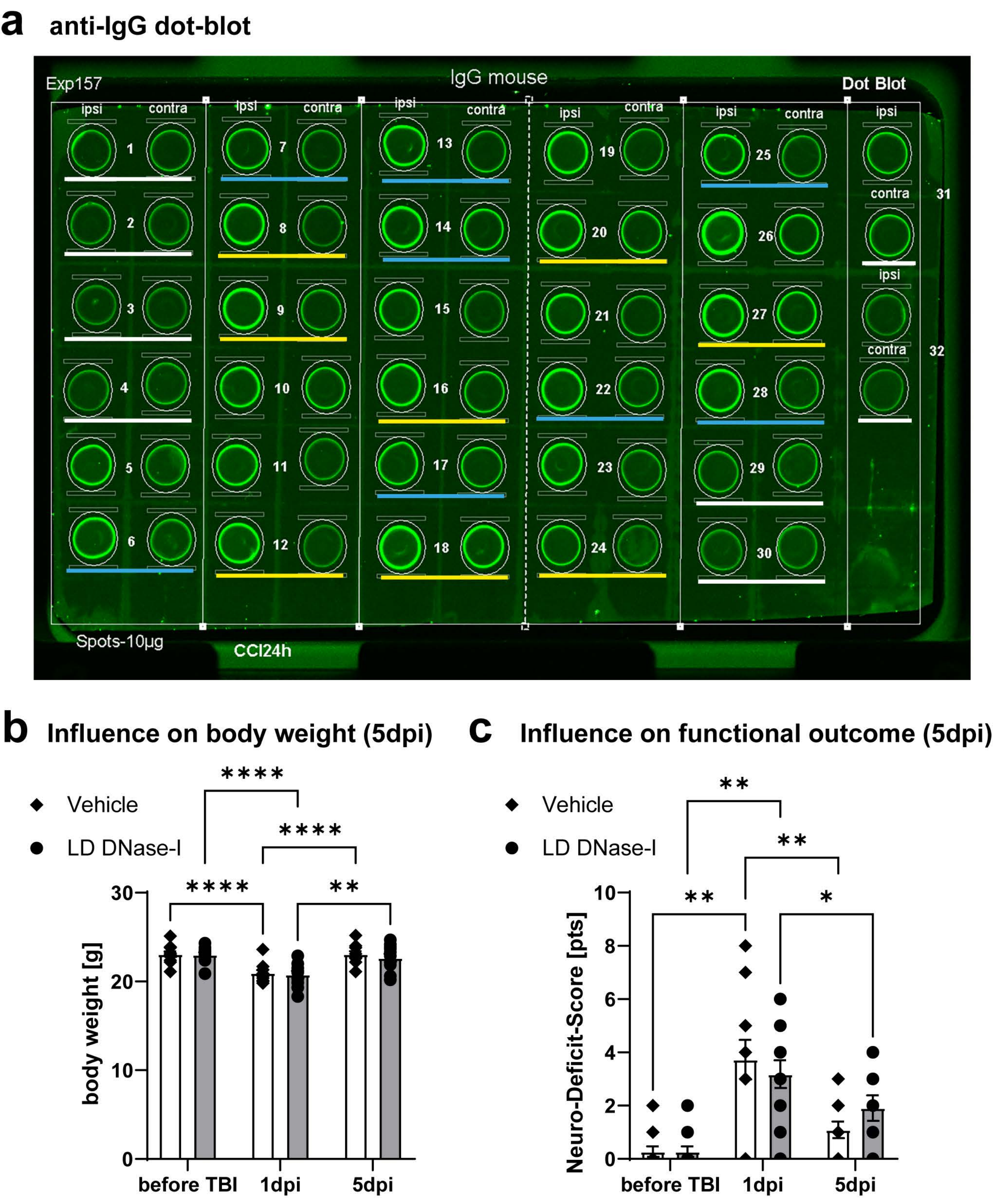

Figure S1: **(a)** Anti-IgG dot-blots with samples from Vehicle (numbers 8, 9, 12, 16, 18, 20, 24 and 27, underlined in yellow color), LD DNase-I (numbers 6, 7, 13, 14, 17, 22, 25 and 28, underlined in blue color) and Sham animals (Numbers 1, 2, 3, 4, 29, 30, 31 and 32, underlined in white color). Quantification of this experiment is shown in Figure 3D. The blot contains additional samples, which are not relevant for the present study (numbers 5, 10, 11, 15, 19, 21, 23, and 26, not underlined). **(b)** Body weight was significantly decreased at 1dpi and recovered at 5dpi, without difference between Vehicle and LD DNase-I treatment groups. **(c)** The Neuro-Deficit-Score was induced after trauma at 1dpi and decreased at 5dpi. No significant differences were observed between Vehicle and LD DNase-I treatment groups.

Figure S2:

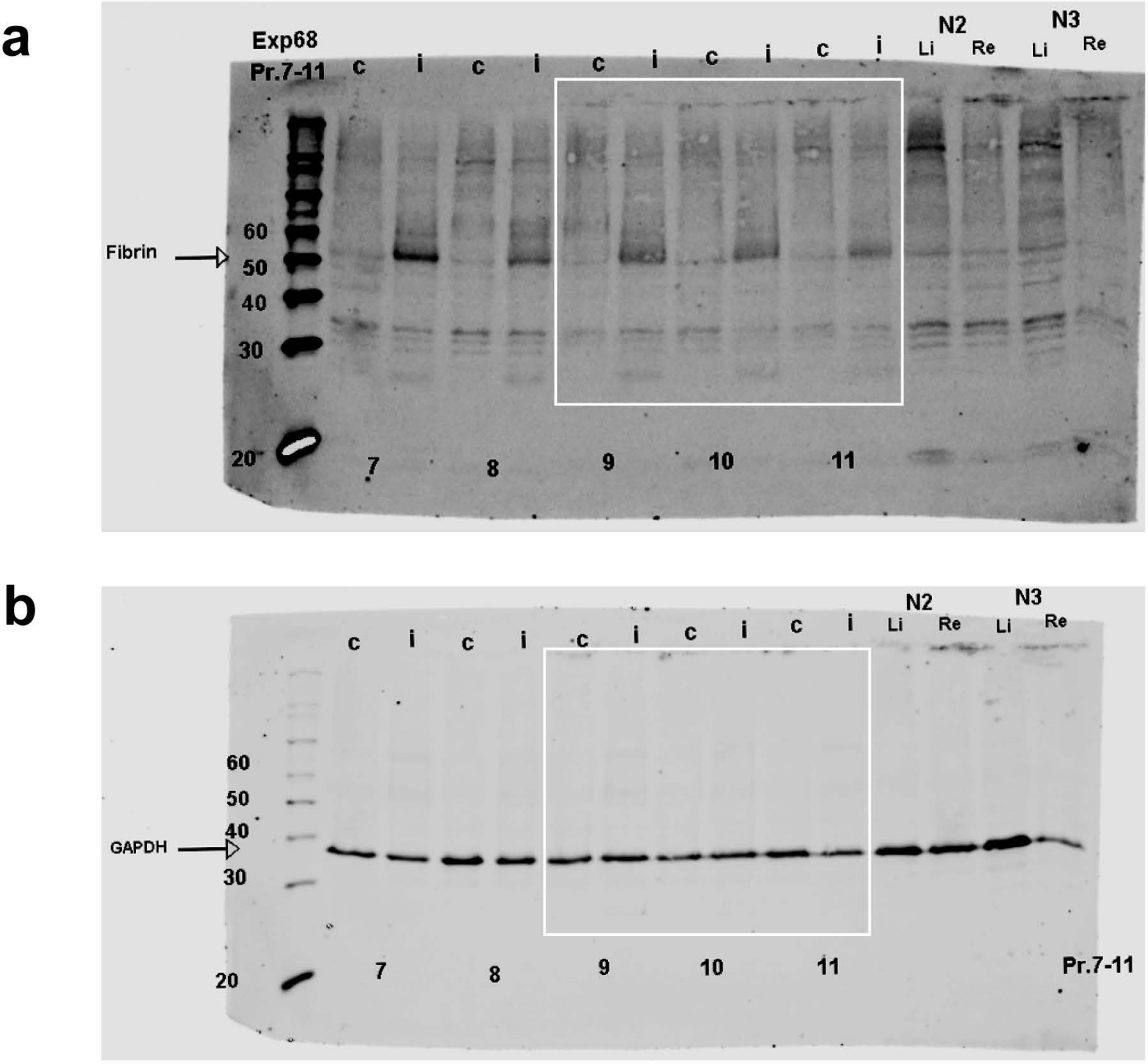

Figure S2: **(a, b)** Western blot membrane probed with antibodies specific for fibrin beta 2 **(a)** or GAPDH **(b)**. The molecular mass marker is shown in the first lane. The framed areas show samples 9-11, presented in Fig. 5b of the article. The area was horizontally flipped for clarity in Fig. 5b. Sample 9: TBI-vehicle, samples 10, 11: TBI-DNase-I, i=ipsilesional, c= contralesional).
